# The Bufei Nashen Pill Alleviates COPD by Targeting Endoplasmic Reticulum Stress Through the PERK/eIF2α Signaling Pathway

**DOI:** 10.1002/iid3.70418

**Published:** 2026-04-13

**Authors:** Tengfei He, Anni Zhang, Changxi Zhang

**Affiliations:** ^1^ Ningxia Medical University Yinchuan China; ^2^ Department of Ophthalmology Beijing Friendship Hospital, Capital Medical University Beijing China; ^3^ Ningxia Hui Autonomous Region Hospital of Traditional Chinese Medicine/ Ningxia Hui Autonomous Region Academy of Traditional Chinese Medicine/ Ningxia Medical University Affiliated Autonomous Region Hospital of Traditional Chinese Medicine Yinchuan China

**Keywords:** Bufei Nashen pill, COPD, endoplasmic reticulum stress, inflammation, PERK/eIF2α signaling pathway

## Abstract

**Background:**

Evidence supporting the therapeutic efficacy of Bufei Nashen pill (BFNSP) for chronic obstructive pulmonary disease (COPD) is currently limited. We evaluated BFNSP's effects on COPD progression and elucidated its potential mechanisms through LC/MS analysis of its active components.

**Methods:**

Following the establishment of a rat COPD model, animals received graded BFNSP doses. Pulmonary function testing quantified respiratory parameters, while HE staining revealed histological changes in lung tissue. ELISA was used to measure TGF‐β1, IL‐6, IL‐8, and IL‐1β concentrations in BALF, as well as MDA, SOD, and GSH levels in lung tissue. Immunofluorescence staining was used to detect the levels of GRP78 and CHOP expression. Protein expression levels of GRP78, CHOP, p‐PERK, p‐eIF2α, and p‐ATF4 in lung tissue were analyzed using Western blotting. The results were further confirmed through in vitro cellular assays.

**Results:**

The study found that BFNSP improved pulmonary ventilation, reduced lung tissue damage, decreased inflammatory factor secretion, and alleviated oxidative and endoplasmic reticulum stress in COPD rats by inhibiting the PERK/eIF2α signaling pathway, potentially slowing COPD progression.

**Conclusion:**

BFNSP mitigates COPD progression by regulating endoplasmic reticulum stress via the suppression of the PERK/eIF2α signaling pathway.

## 1 Introduction

Chronic obstructive pulmonary disease (COPD) is characterized by ongoing and worsening airflow restriction due to sustained airway inflammation. This condition represents a major global health challenge because of its widespread occurrence and serious clinical outcomes [1, 2]. COPD is the third leading cause of death in China, representing a major public health challenge [3]. COPD continues to escalate in both prevalence and lethality, with global annual mortality projected to reach 5.4 million by 2060 [4]. Current pharmacological treatments for COPD encompass corticosteroids, anti‐inflammatory agents, antioxidants, and bronchodilators [5]. Current therapeutic drugs merely alleviate respiratory symptoms or slow disease progression without achieving a cure, demonstrating clear clinical limitations [6]. The urgent need for novel and effective therapeutic approaches remains a critical priority.

In traditional Chinese medicine (TCM), COPD falls under the diagnostic categories of “cough,” “dyspnea,” or “lung distention” disorders [7]. Numerous studies conducted in recent years have demonstrated that TCM has achieved promising outcomes in the management of COPD [8, 9]. Clinical observations reveal that the use of TCM is prevalent among COPD patients, with formulas such as Xiao‐Qing‐Long‐Tang (XQLT) being extensively applied in treatment [10]. TCM provides theoretical benefits and demonstrates substantial efficacy and safety in treating COPD in clinical settings [7]. Consequently, further investigation into the mechanisms of action of TCM on COPD is warranted. As a classical traditional Chinese medicine formula, Bufei Nashen pill (BFNSP) plays a critical role in regulating lung function and mitigating the progression of COPD. Research indicates that BFNSP slows COPD progression by regulating extracellular matrix deposition through the inhibition of the PI3K/AKT/HIF‐1 signaling pathway [11]. BFNSP mitigates lung tissue damage and improves ventilation by suppressing the release of inflammatory factors [11]. The therapeutic effects of BFNSP on COPD and its molecular mechanisms remain poorly understood. Investigating this traditional formula's lung‐kidney tonification properties may advance our understanding of COPD treatment strategies.

The pathological mechanisms underlying COPD are highly complex, with airway inflammation and lung cell apoptosis playing pivotal roles in its progression [12]. Endoplasmic reticulum stress (ERS) significantly contributes to the regulatory mechanisms of inflammation and apoptosis [13]. Research has demonstrated that COPD induces ERS, which in turn is involved in modulating both inflammatory responses and apoptotic processes in COPD [14]. ERS regulates cell survival and death pathways by activating the unfolded protein response (UPR) [15]. During ER stress, GRP78 extensively binds to unfolded or misfolded proteins in the ER lumen, leading to the release of pathway initiation proteins [16, 17]. Following dissociation, PERK undergoes self‐dimerization and phosphorylation, subsequently phosphorylating the downstream eIF2α protein to initiate downstream signaling [18]. Persistent ERS leads to the activation of ATF4 downstream of this pathway, inducing the expression of CHOP, a protein associated with apoptosis, thereby mediating apoptotic processes [19, 20]. In addition, ERS is significantly correlated with oxidative stress in COPD, and this interaction can exacerbate ERS, thereby establishing a vicious cycle that contributes to further lung tissue damage [21]. Consequently, targeted inhibition of ERS may serve as a novel strategy for the development of drugs aimed at preventing and treating COPD.

This study examined the effects of varying doses of BFNSP on COPD model rats by assessing lung function, histopathology, inflammatory markers, the PERK/eIF2α pathway, and ERS factors. The findings were corroborated through in vitro experiments to identify potential therapeutic strategies and targets for COPD.

## 2 Materials and Methods

### 2.1 Establishment of the COPD Model

Forty‐eight 3‐month‐old male SPF‐grade SD rats, weighing 200 ± 20 g, were obtained from Chengdu Dashuo Laboratory Animal Co. Ltd (No. SCXK (Chuan) 2022‐039, Chengdu, China). The rats were provided with food and water without restriction, while ventilation and other environmental conditions were controlled by animal management guidelines. The Experimental Animal Ethics Committee of West China Hospital approved this study (Approval No. 20230625001). After a 1‐week acclimatization, rats were randomly divided into six groups: a control group, a COPD model group, a high‐dose BFNSP group (BFNSP‐H), a medium‐dose BFNSP group (BFNSP‐M), a low‐dose BFNSP group (BFNSP‐L), and a dexamethasone group (DXM). To establish a rat COPD model, the control group was exposed to a combination of cigarette smoke (15 mg of tar, 1.0 mg of nicotine, and 13 mg of carbon monoxide) and lipopolysaccharide (LPS; Sigma, USA) following the methodology outlined in reference [22]. On the 50th day, the Forced Vital Capacity (FVC), Forced Expiratory Volume at 0.1 s (FEV0.1), and Forced Expiratory Volume at 0.3 s (FEV0.3) of the rats in the model group were assessed using a small‐animal pulmonary function testing device (BUXCO, USA). A reduction of more than 30% compared to the control group values was considered indicative of successful COPD modeling.

### 2.2 Method of Administration

After successful modeling, both the control and COPD groups were administered a daily oral gavage of 0.9% NaCl solution at a dosage of 1 mL per 100 g of body weight. The BFNSP‐L, BFNSP‐M, and BFNSP‐H groups received daily gavage doses of BFNSP at 0.125 g/mL, 0.25 g/mL, and 0.5 g/mL per 100 g, respectively. The DXM group received a daily oral gavage of DXM at a concentration of 0.02 mg/mL per 100 g. The drug was administered for a duration of 8 weeks. The BFNSP dosage in this study was determined based on the Pharmacological Laboratory Methodology [23].

### 2.3 Preparation of Cigarette Smoke Extract (CSE)

Preparation of cigarette smoke extract (CSE) involves generating smoke from cigarettes and passing it through a medium to capture the smoke constituents. This extract is then collected for further experimental use. The preparation of CSE is reported in the literature [24]. Cigarette smoke from an unfiltered cigarette is continuously extracted using a negative pressure device and gradually introduced into serum‐free DMEM medium (Procell, Wuhan) to create a uniform CSE solution.

### 2.4 Cell Culture

A549 human alveolar type II epithelial cells (Procell, Wuhan) were cultured in RPMI 1640 medium with 10% fetal bovine serum and 1% antibiotic mixture (Procell, Wuhan) in a 5% CO_2_ incubator. The culture medium was replaced every 2 days, and the cells were passaged once they reached 70%~80% confluency. Subsequently, five groups were formed: the control group (control), the CSE‐exposed group (CSE), the CSE with negative serum group (CSE+CS), the CSE with BFNSP‐containing serum group (CSE+BFNSP‐CS), and the CSE with BFNSP‐containing serum plus PERK activator group (CSE+BFNSP‐CS+CCT020312). Cells were treated with 5% CSE for 24 h, then supplemented with serum containing 5% BFNSP. CCT020312 (HY‐119240, MedChemExpress, Shanghai) was administered to the cells at a concentration of 50 μmol/L. The cells were incubated with 5% CO_2_ for 48 h.

### 2.5 H&E Staining

Lung tissues were fixed in 4% paraformaldehyde, dehydrated with ethanol, cleared using xylene, embedded in paraffin, sectioned, and stained with H&E (Servicebio, Wuhan). The histopathological features of the lung tissues were examined under a microscope (BA210Digital, Motic, Shanghai).

### 2.6 ELISA (Enzyme‐Linked Immunosorbent Assay)

The supernatants of lung tissues, BALF, and A549 cells were collected for subsequent analysis. The levels of MDA, SOD, GSH, TGF‐β1, IL‐6, IL‐8, and IL‐1β were assessed using the ELISA kit from Shanghai Zhuocai Biotechnology, Shanghai, China, according to the manufacturer's instructions.

### 2.7 Immunofluorescence Staining

A549 cells were fixed with 4% paraformaldehyde for 15 min. Subsequently, 0.3% Triton X‐100 was used to permeabilize the cells at room temperature. Subsequently, the samples were incubated for 30 min in PBS with 3% bovine serum albumin (BSA) (GC305010, Servicebio, China) to block non‐specific binding. Primary antibodies, anti‐CHOP (1:200, 15204‐1‐ap, Proteintech, China) and anti‐GRP78 (1:100, R380796, Zenbio, China), were incubated overnight at 4°C. The secondary antibody (1:100, GB23303, Servicebio, China) was added and incubated at room temperature.4′,6‐diamidino‐2‐phenylindole (DAPI) staining solution was applied and incubated for nuclear staining. Finally, the samples were washed, mounted, and covered with a coverslip. Images were captured using a microscopic camera system (OlyVIA, OLYMPUS, Japan). The Image‐J analysis system (National Institutes of Health, USA) was used to calculate the average fluorescence intensity for each image.

### 2.8 Fluo‐4AM Detection for Ca^2+^ Concentration

A549 cells were collected during their logarithmic growth phase. Cells were rinsed with PBS, trypsinized, and collected. Following centrifugation at 250 g for 5 min, the cells were seeded into 6‐well plates with 2 mL per well and incubated at 37°C in a 5% CO₂ environment. After the cells had adhered to the wells, they were grouped and administered with the corresponding treatments. The cells were then collected. The supernatant was removed, and the cells were rinsed with PBS, trypsinized, and centrifuged at 250 g for 5 min. The cell sediment was obtained by discarding the supernatant. The Fluo‐4 AM (S1060, Beyotime, China) stock solution was diluted with PBS to achieve a working concentration of 2.5 μM.1 mL of the working solution was added to the cells, followed by incubation at 37°C for 30 min. After that, the cells were washed three times with PBS, centrifuged at 250 g for 5 min, and the supernatant was discarded to obtain the cell sediment. The cells were resuspended in 300 μL PBS and subjected to analysis via Flow Cytometry (Cytoflex, Beckman Coulter, USA).

### 2.9 Western Blot Analysis

Total protein was extracted from tissues and cells using lysis buffer, followed by a 30‐min lysis on ice to collect cells. Cells were centrifuged at 8000 rpm for 10 min at 4°C to collect the supernatant. Protein concentration was determined by BCA method (P0009, Beyotime, China). The proteins were separated using 10% SDS‐PAGE and blocked with 5% skim milk for 1 h at room temperature. The antibodies used for incubation at 4°C overnight included GRP78 (1:1000, ab21685, Abcam, China), p‐ATF4(Ser245) (1:1000, PA5‐36624, Thermo Fisher, USA), p‐eIF2α(Ser51) (1:2000, ET1603‐14, Huabio, China), p‐PERK(Thr982) (1:1000, 340846, Zenbio, China), CHOP (1:2000, 15204‐1‐AP, Proteintech, China), and β‐actin (1:50000, AC026, ABclonal, China). The sample was washed, incubated with a secondary antibody (1:8000, S0001, Affinity, China) for 1 h, washed again, and then developed. Quantitative evaluation of the results was carried out using Image‐ProPlus 6.0 software.

### 2.10 Statistical Analysis

GraphPad Prism 9.0 was utilized for data analysis. All experimental statistics were expressed using the mean ($\bar{X}$) ± standard deviation (SD). The one‐way analysis of variance (ANOVA) was used to achieve comparative among multiple groups, and statistical analysis was performed post hoc by Tukey. A *t*‐test was conducted to compare the two groups. *p* < 0.05 was statistically significant.

## 3 Results

### 3.1 Impact of BFNSP on Lung Function and Histopathology in COPD

**Figure 1:**
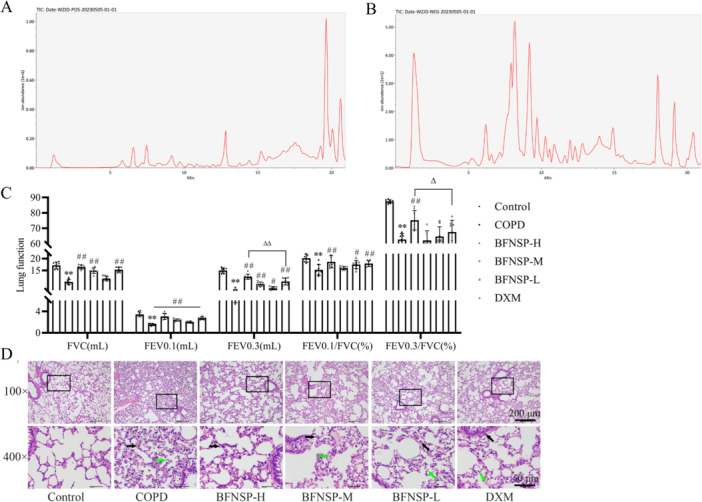
Influence of BFNSP on pulmonary function and histopathological changes in COPD. (A) Chromatograms of positive ions in the BFNSP sample. (B) Chromatograms of negative ions in the BFNSP sample. (C) Levels of FVC, FEV0.1, FEV0.3, FEV0.1/FVC (%), and FEV0.3/FVC (%) in each group of rats. (D) Lung tissue was examined using H&E staining (magnification, 100× and 400×; scale bars, 200 μm and 50 μm; green arrows indicate degenerative necrosis of alveolar epithelial cells, black arrows are neutrophils). The data are expressed as the mean ± SD. Compared with the control group, **p < 0.01; compared with the COPD group, ^#^ *p* < 0.05, ^##^ *p* < 0.01; compared with the DXM group, ^Δ^ *p* < 0.05, ^ΔΔ^ *p* < 0.01.

Firstly, the effective components of BFNSP were systematically analyzed using LC/MS. The LC/MS analysis raw data were transformed into a compatible format using the Analysis Base File Converter software. The formatted raw data were imported into MS‐DIAL 4.70 for preprocessing, encompassing peak extraction, noise reduction, deconvolution, and alignment. The extracted peak information was further compared against established databases, namely MassBank, Respect, and GNPS, to facilitate accurate compound identification. Figure 1 displays the chromatograms of BFNSP in both positive ion mode (Figure 1A) and negative ion mode (Figure 1B). The primary components identified through positive and negative ion chromatographic analysis are detailed in Table S1, providing insights into the pharmacological foundation of BFNSP (Table 1).

**Table 1:** Composition of the BFNSP pill.

Chinese name	Latin name	Family	Dose (g)
Huang qi	*Astragalus membranaceus* Fisch.	Leguminosae	7
Huang jing	*Polygonatum sibiricum* Red.	Liliaceae	8
Rou cong rong	*Cistanche tubulosa* (Schenk) Wight	Cycad	6
Xu duan	*Dipsacus asper* Wall. ex Henry	Dipsacaceae	6
Cang zhu	*Atractylodes lancea* (Thunb.) DC.	Asteraceae	7
Fang feng	*Saposhnikovia divaricata* (Turcz.) Schischk.	Umbelliferae	7
Zi su zi	*Perilla frutescens* (L.) Britt.	Lamiaceae	7
Gua lou	*Trichosanthes kirilowii* Maxim.	Cucurbitaceae	6
Long kui	*Draba nemorosa* L.	Solanaceae	6
Dan shen	*Salvia miltiorrhiza* Bge.	Lamiaceae	7
Gu sui bu	*Drynaria fortunei* (Kunze) J. Sm.	Polypodiaceae	6
Xi xin	*Asarum heterotropoides* F. Schmidt	Aristolochiaceae	5
Zi su ye	*Perilla frutescens* (L.) Britt.	Lamiaceae	6
Jing jie	*Schizonepeta tenuifolia* Briq.	Lamiaceae	6
Can huan mao yin	*Pheretima aspergillum* (E. Perrier)	Megascolecidae	6
Chan tui	*Cryptotympana pustulata Fabricius*	Cicadidae	7
Dong ya qian xie	*Buthus martensii*	Buthidae	2

*Note:* The above formula was prepared in the preparation room of Ningxia TCM Hospital using water pill processing technology and an apparatus containing 3.14 g of raw herbs per gram, which were prepared as Chinese medicine suspensions with distilled water at 100°C before use and divided into 0.125 g/mL, 0.25 g/mL, and 0.5 g/mL solutions according to the high, medium, and low dose concentrations, respectively.

Pulmonary function tests in the COPD group revealed significant reductions in FVC, FEV0.1, FEV0.3, and their respective ratios to FVC (%). In comparison to the COPD group, the BFNSP‐H treatment group exhibited marked improvements in these aforementioned parameters. BFNSP‐H treatment significantly improved FEV0.3 and FEV0.3/FVC (%) compared to DXM treatment (*p* < 0.05, Figure 1C).

H&E staining of lung histopathology in COPD rats showed alveolar epithelial cell shedding, degeneration, necrosis, and varying degrees of inflammatory cell infiltration in the interstitial stroma of the respiratory lung tissue. In the BFNSP‐H group, there was minimal shedding of alveolar epithelial cells into the alveolar space and slight inflammatory cell infiltration in the interstitium (Figure 1D).

### 3.2 Influence of BFNSP on Oxidative Stress and Inflammatory Markers in COPD‐Induced Rats

**Figure 2:**
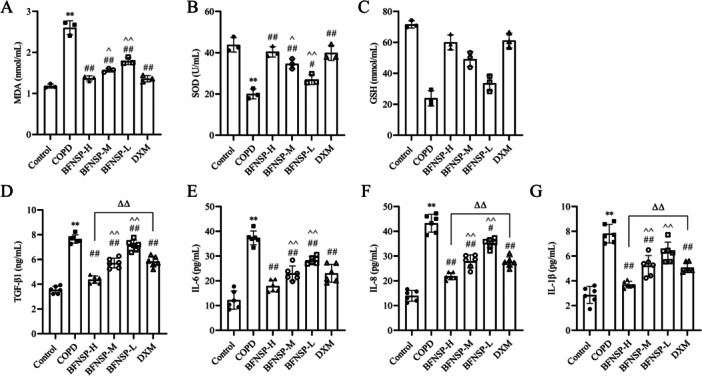
Effect of BFNSP on oxidative stress and inflammatory factor levels in rats with COPD. (A–C): The levels of oxidative stress markers, including MDA, SOD, and GSH, were detected in lung tissue homogenates. (D–G): The concentrations of TGF‐β1, IL‐6, IL‐8, and IL‐1β in BALF were measured by ELISA. The data are expressed as the mean ± SD (*n* = 6). Compared with the control group, **p < 0.01; compared with the COPD group, ^#^ *p* < 0.05, ^##^ *p* < 0.01; compared with the DXM group, ^ΔΔ^ *p* < 0.01; compared with the BFNSP‐H group, ^^^ *p* < 0.05, ^^^^ *p* < 0.01.

The alterations in oxidative stress and inflammatory factor levels in COPD‐induced rats were measured using ELISA, as illustrated in Figure 2. The COPD group showed significantly higher lung tissue MDA levels and notably lower SOD and GSH levels compared to the control group (*p* < 0.01) (Figure 2A–C). Following BFNSP treatment, MDA levels significantly decreased, while SOD and GSH levels notably increased compared to the COPD group (*p* < 0.01). The BFNSP‐H group showed significantly greater efficacy than the BFNSP‐M and BFNSP‐L groups (*p* < 0.05 and *p* < 0.01). In conclusion, BFNSP can alleviate oxidative stress damage in lung tissue and exhibit a beneficial antioxidant effect.

Figure 2D–G shows significantly higher concentrations of TGF‐β1, IL‐6, IL‐8, and IL‐1β in the COPD group than in the control group (*p* < 0.01). Nevertheless, when compared to standard COPD treatment, administration of BFNSP‐H and BFNSP‐M resulted in a substantial reduction in these cytokine levels. BFNSP‐H exhibited a stronger impact than DXM, significantly reducing TGF‐β1, IL‐8, and IL‐1β levels (*p* < 0.01). These findings suggest that BFNSP effectively diminishes inflammatory mediators present in BALF.

### 3.3 Impact of BFNSP on Endoplasmic Reticulum Stress in COPD Rat Lung Tissue

**Figure 3:**
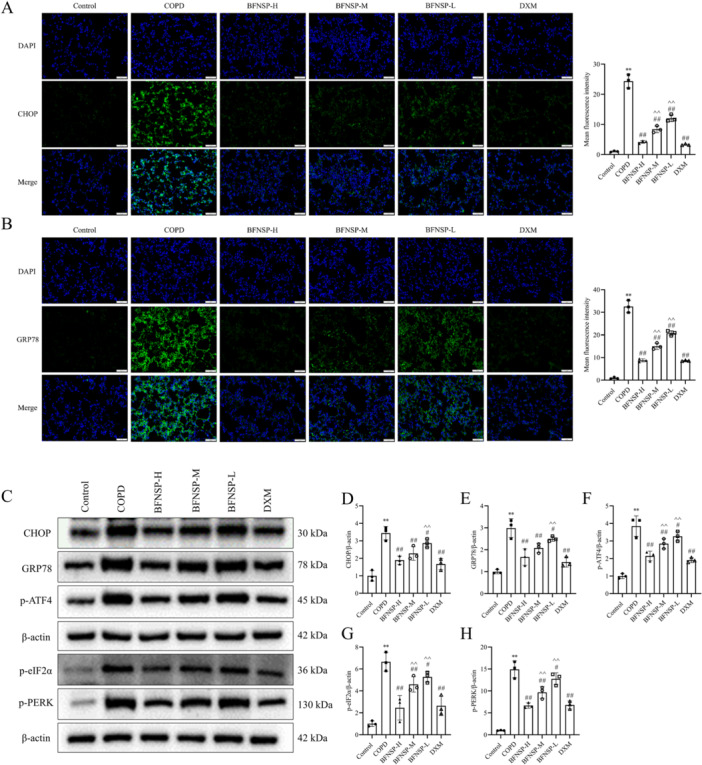
Effect of BFNSP on endoplasmic reticulum stress in lung tissue of COPD rats. (A–B) Representative immunofluorescence images showing the expression of CHOP and GRP78 in lung tissue sections (Magnification: 20X; Scale bar = 50 μm). (C–H) The protein expression levels of ER stress markers (CHOP, GRP78, p‐ATF4, p‐eIF2α, and p‐PERK) were determined by Western blot analysis. Representative Western blot bands. (D–H) Quantitative analysis of relative protein expression. The data are expressed as the mean ± SD (*n* = 6). Compared with the control group, **p < 0.01; compared with the COPD group, ^#^ *p* < 0.05, ^##^ *p* < 0.01; compared with the BFNSP‐H group, ^^^ *p* < 0.05, ^^^^ *p* < 0.01.

Immunofluorescence staining was used to assess the impact of BFNSP on endoplasmic reticulum stress in the lung tissue of COPD rats by measuring the expression levels of related marker proteins. The positive expressions of CHOP and GRP78 were visualized in green. As shown in Figure 3A,B, compared with the control group, the COPD group exhibited significantly higher expression levels of CHOP and GRP78 (*p* < 0.01). After BFNSP treatment, a significant decrease was observed in the expression levels of CHOP and GRP78 when compared to the COPD group, with the BFNSP‐H group demonstrating the most prominent reduction (*p* < 0.01). Western blot analysis was conducted to assess the expression levels of proteins associated with endoplasmic reticulum stress and the PERK signaling pathway (Figure 3C‐H). The COPD group exhibited significantly higher expression levels of CHOP, GRP78, p‐ATF4, p‐eIF2α, and p‐PERK compared to the control group (*p* < 0.01). Conversely, compared to the COPD group, the BFNSP and DXM treatment groups exhibited substantially decreased expression levels of these proteins (*p* < 0.05 and *p* < 0.01) substantially. In contrast to the COPD group, the BFNSP and DXM treatment groups showed significantly lower expression levels of these proteins (*p* < 0.05 and *p* < 0.01). These findings suggested that BFNSP is capable of effectively modulating endoplasmic reticulum stress and suppressing the overactivation of the PERK signaling pathway.

### 3.4 Impact of BFNSP on CSE‐Induced Endoplasmic Reticulum Stress and Inflammatory Factor Expression in A549 Cells

**Figure 4:**
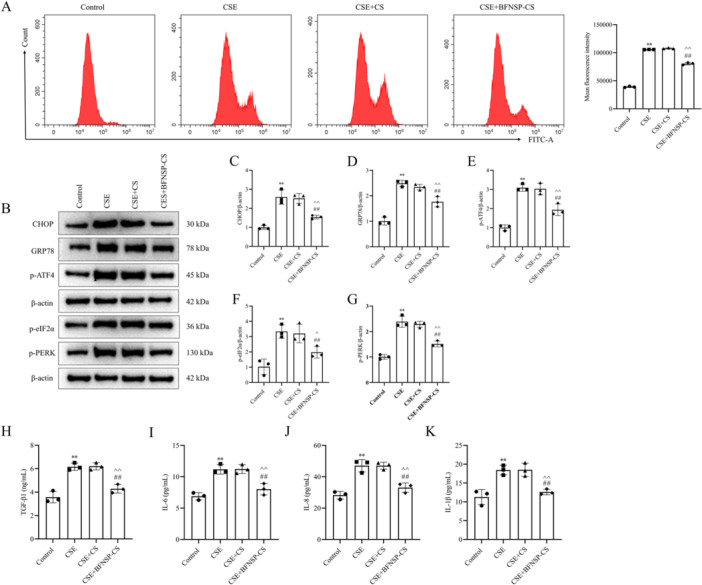
Influence of BFNSP on endoplasmic reticulum stress and inflammatory factor expression induced by CSE in A549 cells. (A) Quantitative flow cytometric analysis of intracellular calcium levels using Fluo‐4 AM staining. (B–G) The protein expression of ER stress markers (CHOP, GRP78, p‐ATF4, p‐eIF2α, and p‐PERK) was analyzed by Western blot. (B) Representative Western blot bands. (C–G) Quantitative analysis of relative protein expression. (H–K) The levels of TGF‐β1, IL‐6, IL‐8, and IL‐1β in A549 cell culture supernatants were detected by ELISA. The data are expressed as the mean ± SD (*n* = 3). Compared with the control group, **p < 0.01; compared with the CSE group, ^#^ *p* < 0.05, ^##^ *p* < 0.01; compared with the CSE‐CS group, ^^^ *p* < 0.05, ^^^^ *p* < 0.01.

The effect of BFNSP on CSE‐induced endoplasmic reticulum stress and inflammatory factors in A549 cells was assessed. First, intracellular calcium levels were detected using Fluo‐4 AM fluorescence (Figure 4A). The CSE group showed a significant increase in Ca^2+^ concentration compared to the control group (*p* < 0.01). Moreover, in comparison to the CES group, a significant reduction in Ca^2+^ levels was observed in A549 cells treated with BFNSP (*p* < 0.01). Western blot analysis indicated a significant upregulation of CHOP, GRP78, p‐ATF4, p‐eIF2α, and p‐PERK expression levels in the CSE group compared to the control group (*p* < 0.01) (Figure 4B‐G). The CSE+BFNSP‐CS group exhibited significantly lower protein expression levels compared to the CSE group (*p* < 0.01). ELISA analysis was performed to quantify cellular inflammatory factors. The study revealed significantly elevated levels of TGF‐β1, IL‐6, IL‐8, and IL‐1β in the CSE group relative to the control group. BFNSP‐CS treatment significantly decreased TGF‐β1, IL‐6, IL‐8, and IL‐1β levels (*p* < 0.05) (Figure 4H‐K).

### 3.5 BFNSP Alleviates Endoplasmic Reticulum Stress in CSE‐Treated A549 Cells Through the PERK/eIF2α Signaling Pathway

**Figure 5:**
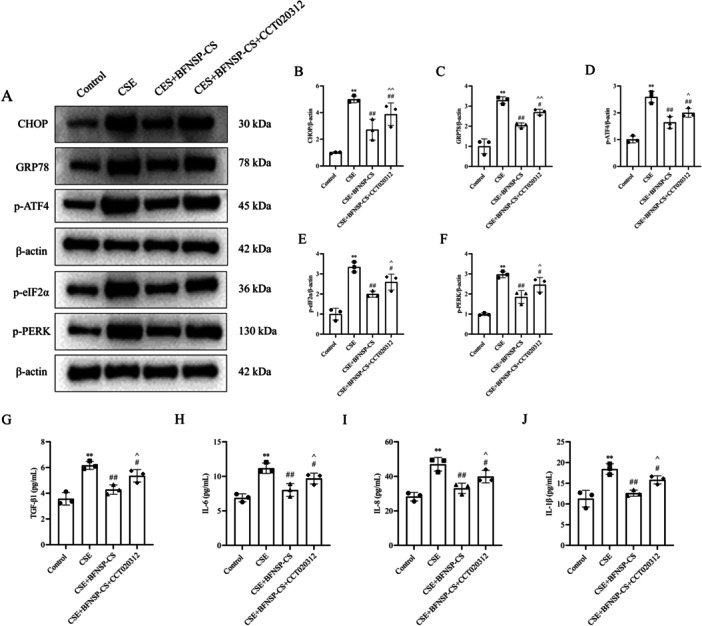
BFNSP inhibits CSE‐treated A549 cells' endoplasmic reticulum stress through the PERK/eIF2α signaling pathway. (A–F) The effect of BFNSP on the PERK/eIF2α signaling pathway was evaluated by Western blot. (A) Representative Western blot bands of CHOP, GRP78, p‐ATF4, p‐eIF2α, and p‐PERK. (B–F) Densitometric analysis of the relative protein expression levels. (G–J) The concentrations of inflammatory cytokines TGF‐β1, IL‐6, IL‐8, and IL‐1β were measured by ELISA. The data are expressed as the mean ± SD (*n* = 3). Compared with the control group, **p < 0.01; compared with the CSE group, ^#^ *p* < 0.05, ^##^ *p* < 0.01; compared with the CSE+BFNSP‐H group, ^^^ *p* < 0.05, ^^^^ *p* < 0.01.

CCT020312 is a selective PERK activator, as shown in Figure 5. The CSE+BFNSP‐CS+CCT020312 group exhibited significantly elevated protein expression of CHOP, GRP78, p‐ATF4, p‐eIF2α, and p‐PERK (Figure 5A‐F), as well as increased levels of TGF‐β1, IL‐6, IL‐8, and IL‐1β (Figure 5G‐J), compared to the CSE+BFNSP‐CS group (*p* < 0.05 and *p* < 0.01). CCT020312 counteracted BFNSP's suppression of endoplasmic reticulum stress and inflammation. These findings indicate that BFNSP may mitigate endoplasmic reticulum stress and inflammation by inhibiting the PERK/eIF2α signaling pathway.

## 4 Discussion

Analysis of BFNSP components identified several primary compounds with potential therapeutic effects against COPD. Rosmarinic acid exhibited strong anti‐inflammatory properties in lung tissues [25]. Pristimerin exhibited strong anti‐inflammatory properties by inhibiting NF‐κB‐mediated cytokine production, highlighting its potential in treating inflammatory lung diseases such as COPD [26]. Tuberostemonine exhibited anti‐inflammatory properties in a cigarette smoke‐induced acute lung inflammation model [27]. Theophylline exhibits anti‐inflammatory properties in COPD [28]. Furthermore, theophylline has been found to reverse the steroid insensitivity in COPD patients, which is related to oxidative stress [29]. These findings establish a pharmacodynamic mechanism underlying BFNSP's therapeutic effects in COPD. Future work will characterize the primary bioactive constituents through in vitro assays and animal models.

COPD is a progressive respiratory condition characterized by ongoing airflow limitation and chronic lung inflammation [30]. This study developed a COPD rat model using a combination of smoke exposure and LPS administration. This approach significantly shortened the modeling duration while reducing mortality rates and more closely mimicking the natural progression of COPD pathogenesis [31]. Following model establishment, pulmonary function tests revealed airflow limitation in the ventilatory parameters, confirming successful COPD rat model construction. COPD is marked by ongoing chronic inflammation impacting the lungs and showing systemic effects [32]. Cytokines such as TNF‐α, IL‐1, and IL‐6 play a crucial role in the inflammation associated with COPD. Their overexpression is strongly associated with disease severity [33, 34]. The study found marked increases in IL‐6, IL‐8, IL‐1β, and TGF‐β1 levels in lung tissue and BALF, highlighting a significant inflammatory response in rat lungs. These findings are consistent with those reported by ZHANG et al. [35]. Pathological analysis revealed distinct structural changes in the lung tissue of COPD rats, including alveolar wall thickening and inflammatory cell infiltration. These pathological alterations align with the clinical observations reported by WANG et al. [36]. Following BFNSP treatment, COPD rats exhibited marked improvements in pulmonary ventilation function and pathological features, along with significant suppression of inflammatory responses, indicating the BFNSP anti‐inflammatory efficacy against COPD.

Recently, the role of endoplasmic reticulum stress (ERS) in COPD pathogenesis has garnered increasing attention. Numerous studies highlight ERS as a crucial factor in early COPD development and its significant role in inducing apoptosis in alveolar epithelial cells [37, 38, 39]. Cigarette smoke (CS), the main risk factor for pulmonary inflammation in COPD patients, produces active intermediates that cause oxidative damage and protein misfolding in the lungs. Over time, the accumulation of these misfolded proteins leads to endoplasmic reticulum stress (ERS) [40]. Kelsen et al. Proteomic analysis demonstrated that extended smoking leads to increased expression of various UPR chaperone proteins and folding enzymes in human lung tissue [41]. Studies have shown a significant increase in the expression levels of CHOP and GRP78 proteins and their mRNAs in the lung tissues of rats with COPD [42, 43]. Extended CS exposure increases GRP78, ATF4, ATF6, and CHOP protein expression in mouse lung tissue and enhances PERK and IRE1 phosphorylation [39]. Notably, Kenche et al. A single cigarette exposure can increase phosphorylated eIF2α and ATF6 (p50) levels in mouse lung tissue [44]. This study examined the expression levels of oxidative stress markers (MDA, SOD, and GSH) and endoplasmic reticulum stress‐related proteins (GRP78 and CHOP) in the lung tissues of rats with COPD. The results showed significantly elevated levels of MDA, SOD, GSH, GRP78, and CHOP in COPD rats. BFNSP treatment significantly reduced these markers, indicating its capacity to mitigate COPD progression through endoplasmic reticulum stress modulation. Furthermore, in vitro experiments revealed that CSE stimulation significantly increased the levels of GRP78 and CHOP in A549 cells, whereas BFNSP‐CS markedly reduced these levels, indicating that CSE induces epithelial cell damage and BFNSP‐CS mitigates such damage.

PERK is a type I transmembrane protein kinase located on the endoplasmic reticulum membrane. It becomes activated via autophosphorylation and homodimerization after disengaging from GRP78 [16]. Upon activation, PERK phosphorylates eIF2α, reducing global translation and protein synthesis [45]. Nevertheless, the phosphorylation of eIF2α promotes the translation of mRNAs that contain upstream open reading frames, including those encoding transcription factor ATF4 [46]. ATF4 functions as a stress‐responsive transcription factor that induces apoptosis by activating C/EBP homologous protein (CHOP) transcription under prolonged stress. Moreover, the phosphorylation of eIF2α plays a role in regulating the translation of other proteins associated with endoplasmic reticulum stress, including growth arrest‐specific proteins [47]. In conclusion, PERK activation alleviates protein load in the endoplasmic reticulum, but if homeostasis is not restored, it may trigger cell death pathways. In COPD rat lung tissues, there was a notable increase in p‐PERK, p‐eIF2α, and p‐ATF4 expression, indicating activation of the PERK/eIF2α pathway. BFNSP reduced the levels of these phosphorylated proteins, effectively suppressing PERK/eIF2α signaling. Moreover, BFNSP‐CS effectively suppressed the activation of the PERK/eIF2α pathway in CSE‐induced cells.

The unfolded protein response (UPR) is crucial for cellular adaptation to endoplasmic reticulum (ER) stress. It not only aids in restoring protein homeostasis but is also intricately linked to the modulation of inflammatory responses. Research has demonstrated that UPR activation can notably enhance the production of low‐level inflammatory cytokines. Furthermore, the three core UPR sensors PERK, IRE1, and ATF6 each contribute to the regulation of the inflammatory process via distinct molecular mechanisms under ER stress conditions. In the CS‐induced COPD animal model, lung tissue showed a marked ERS response. Furthermore, bronchoalveolar lavage fluid analysis revealed a notable rise in both the total inflammatory cell count and neutrophil percentage. Inflammation‐associated factors, including IL‐6, IL‐8, and TNF‐α, showed significantly increased levels [42]. Compounds capable of inhibiting or blocking the ER stress signaling pathway not only mitigate CS‐induced airway inflammation but also contribute positively to the alleviation of emphysema [48]. This study revealed that inflammatory factor expression levels in the BALF of COPD rats were markedly elevated compared to the control group. BFNSP treatment significantly reduced their expression, indicating its potential to slow COPD progression by modulating inflammatory responses. CSE‐stimulated A549 cells showed a marked rise in inflammatory mediator levels in vitro. However, treatment with BFNSP‐CS markedly reduced their expression, further indicating its potential to alleviate CSE‐induced inflammation. CCT020312 is a selective activator of PERK. The study found that co‐administering BFNSP‐CS and CCT020312 significantly increased the levels of TGF‐β1, IL‐6, IL‐8, and IL‐1β, along with the expression of CHOP, GRP78, p‐PERK, p‐eIF2α, and p‐ATF4 in cells. These findings indicated that CCT020312 effectively reversed the inhibitory effects of BFNSP‐CS on ER stress and inflammation. This indicates that BFNSP‐CS might mitigate ER stress and inflammation by inhibiting the PERK/eIF2α pathway.

## 5 Conclusion

In summary, our findings demonstrated that BFNSP improves pathological damage in COPD lung tissue, suppresses the release of inflammatory mediators, and mitigates oxidative stress and endoplasmic reticulum stress. This effect may be associated with BFNSP's ability to inhibit the activation of the PERK/eIF2α signaling pathway. These research findings may provide a robust basis for the advancement of COPD therapeutic strategies. However, the specific active components of BFNSP should be tested in future COPD models.

## Author Contributions

**Tengfei He:** software, validation, visualization, writing – original draft, writing – review and editing. **Anni Zhang:** conceptualization, methodology, software, validation, investigation, visualization. **Changxi Zhang:** formal analysis, data curation, resources, writing – review and editing, supervision, project administration, funding acquisition. All authors have read and agreed to the published version of the manuscript.

## Ethics Statement

This study involving vertebrates (rats) was approved by the Experimental Animal Ethics Committee of West China Hospital (Approval No.: 20230625001). All procedures adhered to relevant ethical guidelines and regulations to ensure animal welfare and minimize suffering.

## Conflicts of Interest

The authors declare that they have no known competing financial interests or personal relationships that could be perceived as exerting influence on the findings presented in this paper.

## Supporting information

Supplement‐Table S1.

## Data Availability

All the data is fully available without any restrictions.
